# The Main Structural Unit Elucidation and Immunomodulatory Activity In Vitro of a Selenium-Enriched Polysaccharide Produced by *Pleurotus ostreatus*

**DOI:** 10.3390/molecules27082591

**Published:** 2022-04-18

**Authors:** De Wang, Jiahui Wang, Hui Liu, Meng Liu, Yanjing Yang, Shian Zhong

**Affiliations:** College of Chemistry and Chemical Engineering, Central South University, Changsha 410083, China; trpasp@163.com (D.W.); wangjiahui4869@163.com (J.W.); liu18390955104@163.com (H.L.); lm_csu@163.com (M.L.)

**Keywords:** *Pleurotus ostreatus*, selenium-enriched polysaccharides, immunomodulatory activity

## Abstract

In recent years, the structure of selenium-enriched polysaccharides and their application in immunomodulation have attracted much attention. In previous studies, we extracted and purified a novel selenium-enriched *Pleurotus ostreatus* polysaccharide called Se-POP-21, but its structure and immunomodulatory activity were still unclear. In this study, the main structural unit formula of Se-POP-21 was characterized by methylation analysis and an NMR experiment. The results showed that the backbone of Se-POP-21 was →[2,6)-α-D-Galp-(1→6)-α-D-Galp-(1]_4_→2,4)-β-L-Arap-(1→[2,6)-α-D-Galp-(1→6)-α-D-Galp-(1]_4_→, branched chain of β-D-Manp-(1→ and β-D-Manp-(1→4)-β-L-Arap-(1→ connected with →2,6)-α-D-Galp-(1→ and →2,4)-β-L-Arap-(1→,respectively, through the O-2 bond. In vitro cell experiments indicated that Se-POP-21 could significantly enhance the proliferation and phagocytosis of RAW264.7 cells, upregulate the expression of costimulatory molecules CD80/CD86, and promote RAW264.7 cells to secrete NO, ROS, TNF-α, IL-1β, and IL-6 by activating the NF-κB protein. The results of this study indicate that Se-POP-21 can effectively activate RAW264.7 cells. Thus, it has the potential to be used in immunomodulatory drugs or functional foods.

## 1. Introduction

The body’s immune system can recognize native and foreign substances and eliminate antigenic foreign bodies through the immune response, thus maintaining the body’s physiological balance [1]. Specifically, the immune system uses PRRs (pattern recognition receptors) to recognize specific molecular signatures on pathogens called PAMPs (pathogen-associated molecular patterns). The recognition of PAMPs by PRRs leads to antigen-presenting cell activation and elicits adaptive immunity [2,3]. Therefore, the immune system is an essential defensive system for the body to produce an immune response and protect itself [4]. In recent years, effective regulation of the immune system has gradually become the focus of research [5].

Polysaccharides are a class of macromolecular active substances that widely exist in organisms, and consist of multiple monosaccharides linked by glycosidic bonds. They have a variety of physiological activities, such as antitumor, immunomodulatory, antioxidant, etc. [6,7]. Many polysaccharides have been proven to play an immunomodulatory role by activating immune cells and promoting the secretion of cytokines and antibodies [8,9,10].

Studies in recent years have shown that selenium-enriched polysaccharides have better immunomodulatory activity than selenium or polysaccharides alone, and they can improve the bioavailability of selenium so that the human body can quickly absorb it [11,12]. For example, selenium *Chuanminshen violaceum* polysaccharides can significantly promote the proliferation of spleen cells, improve the production of IL-4 and IFN-γ, and increase the cytotoxicity of natural killer cells and T-lymphocyte activity in vitro [13]. Selenium *Hericium Erinaceus* polysaccharides can promote the proliferation of macrophages and increase the secretion of NO, TNF-α, IL-1β, and IL-6 by macrophages. These results suggest that selenium *Hericium Erinaceus* polysaccharides may induce a robust immune response by enhancing the activation of macrophages [14].

Macrophages are crucial immune cells in the body, with a wide range of physiological functions [15]. Activated macrophages can phagocytose various pathogenic microorganisms, process and present antigens, and synthesize and secrete various cytokines, thus enhancing the body’s immunity [16,17]. Therefore, macrophages have been used as cell models in vitro to study the immunomodulatory effects of polysaccharides [14]. 

In addition, a review of the literature shows that the immunomodulatory activity of polysaccharides seems to be related to their structure—especially the composition of monosaccharides [18,19,20]. Lo et al. [21] used multiple linear regression analysis to speculate on the correlation between the monosaccharide composition of *Lentinus edodes* polysaccharides and their macrophage-stimulating activity in vitro. The results indicate that arabinose, mannose, galactose, and xylose may be related to the macrophage-stimulating activity of polysaccharides. Our previous study extracted and purified a new type of selenium-enriched *Pleurotus ostreatus* polysaccharide called Se-POP-21, principally composed of galactose, mannose, arabinose, and glucose, at a molar ratio of 26.15:18.01:7.34:2.40, respectively [22]. According to the above literature reports, Se-POP-21 is likely to have potential macrophage-stimulating activity. In addition, the selenium-enriched *Pleurotus ostreatus* is the best-selling and most economical selenium-enriched functional food on the market [23]. However, there are no reports on the immunomodulatory activity of selenium-enriched *Pleurotus ostreatus*.

Therefore, it is of solid theoretical basis and practical significance to study the structure and immunomodulatory activity of selenium-enriched *Pleurotus ostreatus* polysaccharides. This study determined the primary structural unit formulae of Se-POP-21 via methylation analysis and NMR experiments. The immunoregulatory activity of Se-POP-21 in vitro was also studied using the RAW264.7 cell model. In summary, this study makes preliminary explorations to elucidate the relationship between the structure of Se-POP-21 and its immunomodulatory activity. It may provide a theoretical basis for applying selenium-enriched *Pleurotus ostreatus* polysaccharides in immunomodulatory drugs or functional foods.

## 2. Results and Discussion

### 2.1. Methylation Analysis 

The retention time and the mass spectrograms of PMAA were used to identify the glycosylation linkage of Se-POP-21 [24] (the mass spectrograms of PMAA are shown in the Supplementary Materials; Se-POP-21 was fully reacted to become PMAA). As shown in Table 1, according to the results of the methylation analysis of Se-POP-21, there were seven types of methylated sugars in Se-POP-21: 2,3,4,6-Me_4_-Manp, 2,3,4,6-Me_4_-Galp, 2,4-Me_2_-Arap, 2,3-Me_2_-Arap, 2,3,4,-Me_3_-Galp, 3-Me-Arap, and 3,4,-Me_2_-Galp, at a ratio of 0.325: 0.012: 0.016: 0.041: 0.280: 0.039: 0.287, respectively. The corresponding linkage types were Manp-(1→, Galp-(1→, →3)-Arap-(1→, →4)-Arap-(1→, →6)-Galp-(1→, →2,4)-Arap-(1→, →2,6)-Galp-(1→. It is worth noting that, combined with the methylation analysis results and the monosaccharide composition results obtained in previous studies, the galactose-based glycosylic bond constitutes the main skeleton of Se-POP-21. However, the glucose content is too small to be detected. 

### 2.2. Analysis of NMR

**Figure 1:**
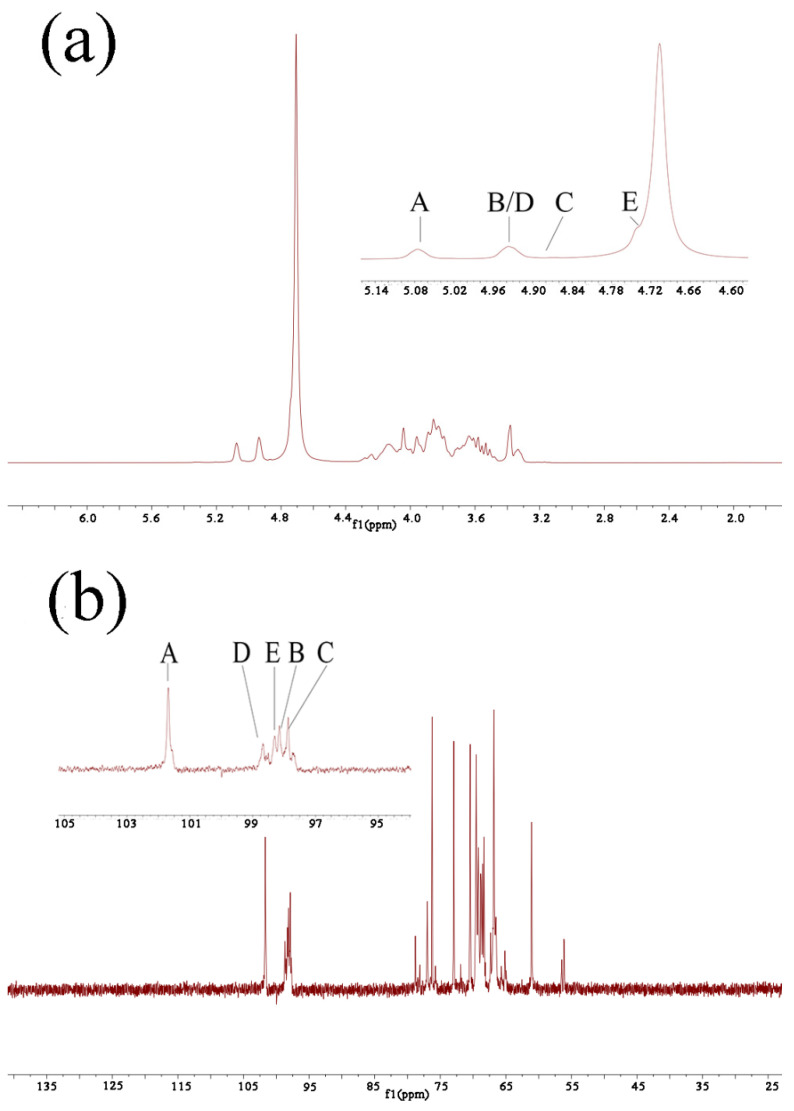

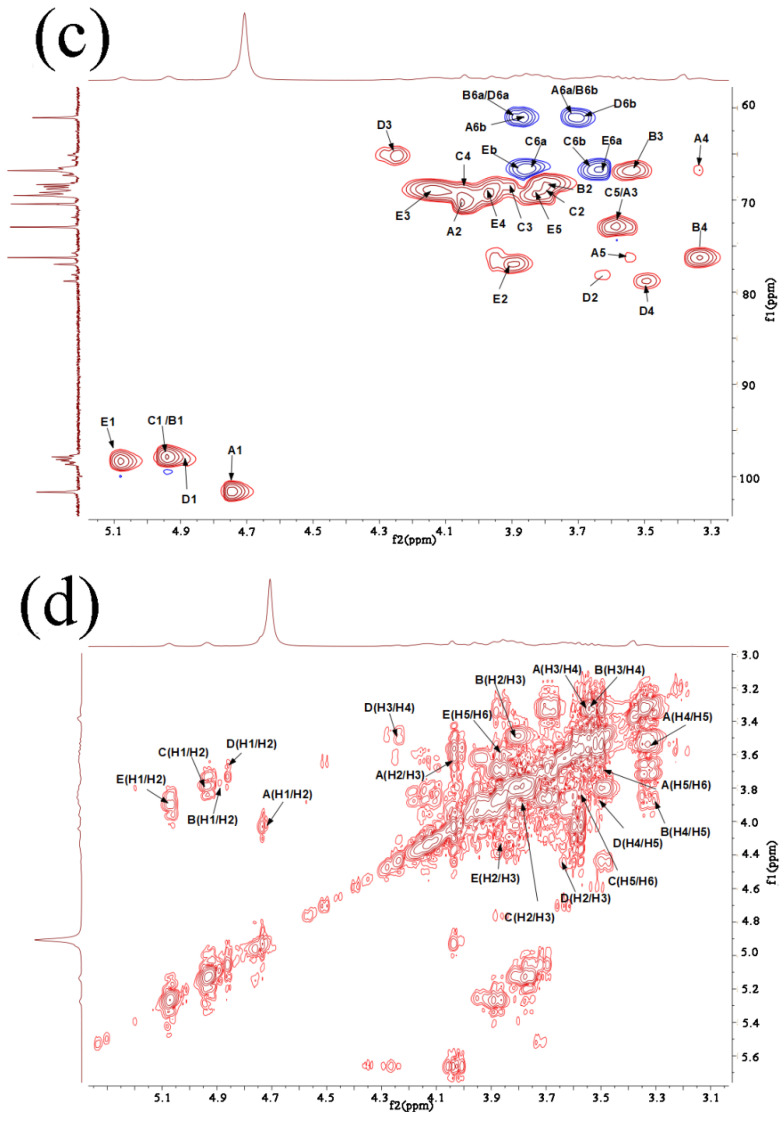

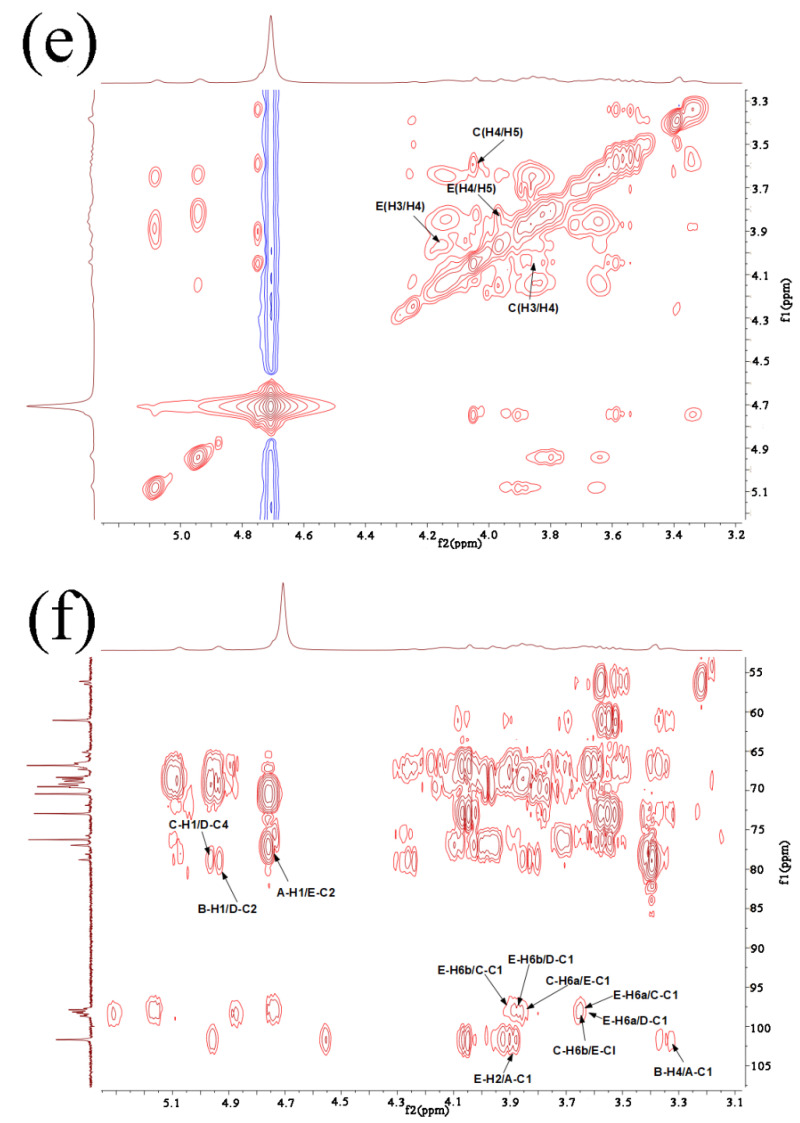
^1^H NMR (**a**), ^13^C NMR (**b)**, HSQC (**c**), COSY (**d**), NOESY (**e**), and HMBC (**f**) spectra of Se-POP-21. A–E in Figure (**a**) and (**b**) are the signal peaks of anomeric hydrogen and anomeric carbon, respectively.

**Figure 2:**
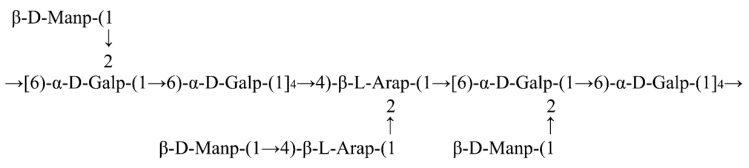
The main structural unit formula of Se-POP-21.

However, methylation analysis could not determine the binding sequence between sugar residues, and the introduction of NMR technology makes up for this deficiency [25]. One-dimensional NMR spectra mainly include the ^1^H NMR spectrum and ^13^C NMR spectrum. The ^1^H NMR spectrum could provide the hydrogen signals of anomeric hydrogen and other positions of the sugar ring, which is helpful to analyze the configuration of the glycoside bond. However, the complexity of the polysaccharide structure leads to the serious overlap of the hydrogen spectrum signals. In contrast, the ^13^C NMR spectrum has a wider range of chemical shifts and less spectral line overlap [26,27]. The number of sugar residues could be inferred from anomeric carbons in the ^13^C NMR spectrum. Then, H-H coupling information, C-H coupling information, and the connection sequence of glycosidic bonds were deduced from the COSY, HSQC, NOESY, and HMBC spectra to determine the main structural unit formula of Se-POP-21 [28,29,30]. As shown in the ^1^H NMR spectrum (Figure 1A), the peaks at δ4.74, 4.89, 4.94, 4.86, and 5.06 were the signals of the anomeric proton, and the other proton signals of the sugar residues were located at δ3.2–4.2. In particular, the signal at δ4.74 was covered by the D_2_O peak, which could be inferred from the HMBC spectrum. The ^13^C NMR spectrum (Figure 1B) showed that the peaks at δ101.62, 98.15, 97.66, 98.68, and 98.34 were the main anomeric carbon signals, and the other carbon signals of the sugar residues were located between δ60 and δ80 [26,31]. Then, combining the HSQC (Figure 1C) and COSY (Figure 1D) spectra, we can see the anomeric carbon signals at δ101.62, 98.15, 97.66, 98.68, and 98.34, corresponding to the anomeric proton signals at δ4.74, 4.89, 4.94, 4.86, and 5.06, respectively. This indicates that there were five main sugar residues, named A, B, C, D, and E. According to the anomeric proton signals, it can be inferred that the residues C and E are in the α configuration, while the residues A, B, and D are in the β configuration [32]. Next, starting from the anomeric carbon, each sugar residue’s ^13^C and ^1^H signals were inferred from the COSY, NOESY (Figure 1E), and HSQC spectra. Taking residue A as an example, taking the anomeric proton signal at δ4.74 as a breakthrough point and combining it with the COSY and NOESY spectra, it can be concluded that the signals of H2, H3, H4, H5, H6a, and H6b are at δ4.03, 3.56, 3.33, 3.54, 3.70, and 3.87, respectively. Similarly, combining COSY, NOESY, and HSQC spectra, it can be concluded that the signals of C2, C3, C4, C5, and C6 are at δ70.32, 72.55, 66.78, 76.21, and 61.07, respectively. According to the methylation results and the standard monosaccharide ^13^C NMR signal data, residue A was intended to be β-D-Manp-(1→. In the same way, residues B, C, D, and E were determined to be →4)-β-L-Arap-(1→, →6)- α-D-Galp-(1→, →2,4)-β-L-Arap-(1→ and →2,6)-α-D-Galp-(1→, respectively. The chemical shifts of ^1^H and ^13^C of residues A, B, C, D, and E, along with the corresponding analysis results, are shown in Table 2. The content of other residues was too low to be effectively assigned. The connection mode between the residues mentioned above can be shown by the HMBC (Figure 1F) spectrum. As shown in the HMBC spectrum, the anomeric carbon of residue C and the H6a/H6b of residue E have a correlation signal peak, indicating that→6)-α-D-Galp-(1→ and →2,6)-α-D-Galp-(1→were linked. The anomeric carbon of residue E and the H6a/H6b of residue C also have a correlation signal peak, indicating that →2,6)-α-D-Galp-(1→ and →6)-α-D-Galp-(1→were linked. The above results indicate that residues C and E have a head-to-tail link. Furthermore, the anomeric carbon of residue D and the H6a/H6b of residue E have a correlation signal peak, indicating that →2,4)-β-L-Arap-(1→ and →2,6)- α-D-Galp-(1→ were linked. The anomeric proton of residue C and the C4 of residue D have a correlation signal peak, indicating that→6)-α-D-Galp-(1→and →2,4)-β-L-Arap-(1→ were linked. The above analysis shows that residues C, D, and E are linked to one another, and constitute the host chain of the Se-POP-21 structure. Similarly, the anomeric proton of residue A and the C2 of residue E have a correlation signal peak, indicating that β-D-Manp-(1→ and →2,6)-α-D-Galp-(1→were linked. The anomeric proton of residue B and the C2 of residue D have a correlation signal peak, indicating that →4)-β-L-Arap-(1→ and →2,4)-β-L-Arap-(1→ were linked. According to the relative content of each residue, there are some repeating units in the structure of Se-POP-21. Based on the above analysis results, the main structural unit formula of Se-POP-21 is shown in Figure 2. Due to the low selenium content, this study cannot determine its specific location in Se-POP-21, so Figure 2 does not reflect the existence of selenium. According to the FTIR spectra obtained from previous studies [22], it can be inferred that selenium may be linked to Se-POP-21 in the form of selenious acid through the Se-O-C bond.

### 2.3. Effects on RAW264.7 Cells’ Viability

**Figure 3:**
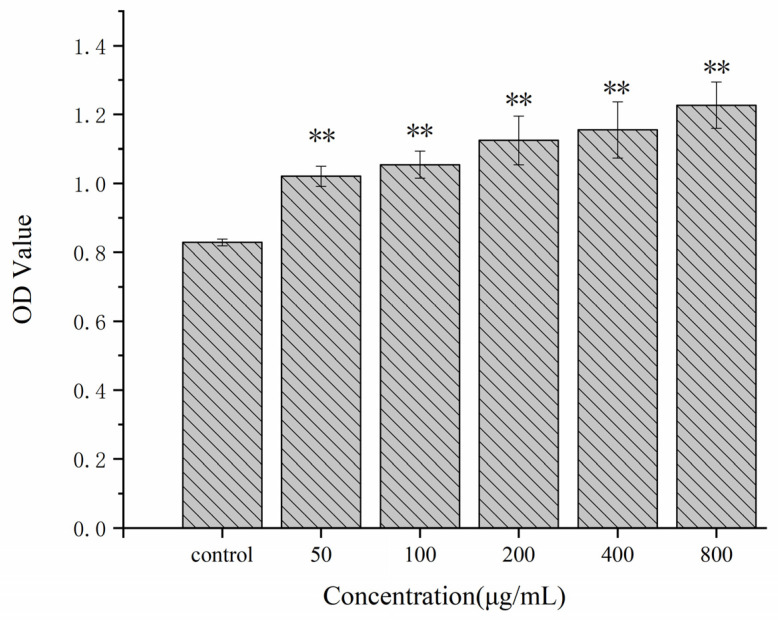
Effects of Se-POP-21 on RAW264.7 cells’ viability. The data are expressed as mean ± SD; ** *p* < 0.01 compared with the control group.

In this study, the CCK-8 assay was used to evaluate the effects of various concentrations of Se-POP-21 on RAW264.7 cells’ viability. As shown in Figure 3, compared with the blank control group (the group without Se-POP-21), the OD value of RAW264.7 cells incubated with Se-POP-21 at various concentrations (0, 50, 100, 200, 400, 800 μg/mL) was significantly increased in a dose-dependent manner. This indicates that Se-POP-21 could promote the proliferation of RAW264.7 cells. The effect of Se-POP-21 on the proliferation of RAW264.7 cells was significantly greater than that of *Psoralea corylifolia* polysaccharides at the same concentrations [33]. At the same time, this result also suggests that Se-POP-21 has no cytotoxicity within the above concentration range. Therefore, Se-POP-21 at concentrations of 200, 400, and 800 μg/mL was selected for subsequent experiments to obtain the desired effect.

### 2.4. Effect on Phagocytosis of RAW 264.7 Cells

**Figure 4:**
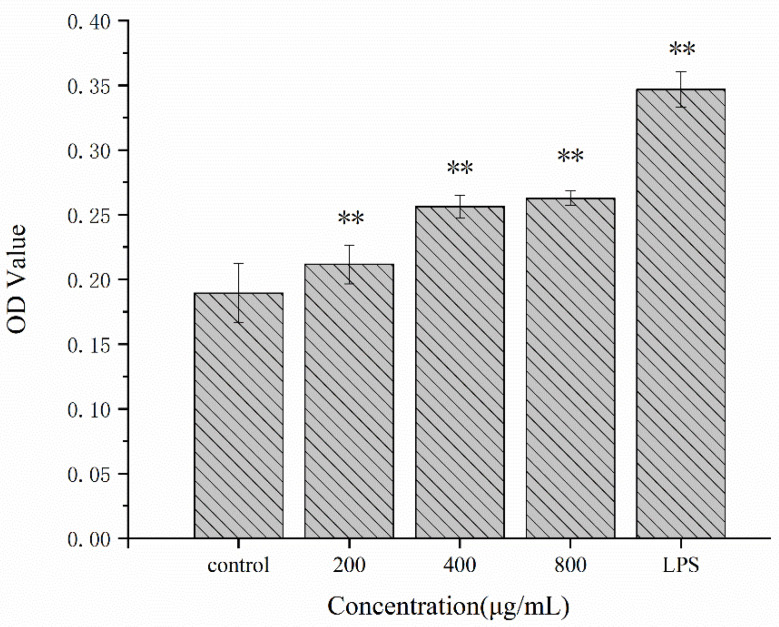
Effects of Se-POP-21 on the phagocytosis of RAW 264.7 cells. The data are expressed as mean ± SD; ** *p* < 0.01 compared with the control group.

The phagocytosis of macrophages is a vital part of the nonspecific immune response. Macrophages can swallow pathogenic microorganisms and digest them [34]. Meanwhile, the capacity for phagocytosis can also reflect the activity of macrophages [35]. In this study, the neutral red assay was used to investigate the effects of different concentrations of Se-POP-21 on the phagocytosis of RAW264.7 cells. The OD value of the neutral red assay could reflect the phagocytic ability of the RAW264.7 cells. Lipopolysaccharide (LPS) is a classical and potent immune-cell activator, and is a recognized positive control for immune experiments. Therefore, in this study, LPS was used as a positive control to compare the immune activation of Se-POP-21. As shown in Figure 4, compared with the blank control group, the OD value of RAW264.7 cells treated with Se-POP-21 at different concentrations (200, 400, 800 μg/mL) was significantly increased. This result indicates that Se-POP-21 could improve the phagocytosis capacity of RAW264.7 cells, thus promoting the nonspecific immune function of RAW264.7 cells. As there is a large amount of mannose at the end of Se-POP21, it may bind to mannose receptors (MRs) on the surface of RAW264.7 and activate the NF-κB pathway to activate RAW264.7 cells, thus enhancing the phagocytosis of cells. In addition, according to the literature, activated RAW264.7 cells release cytokines such as NO, TNF-α, and IL-1β, which can synergistically enhance cell phagocytosis [36,37]. 

### 2.5. Effects on Phenotypic Alterations of RAW264.7 Cells

**Figure 5:**
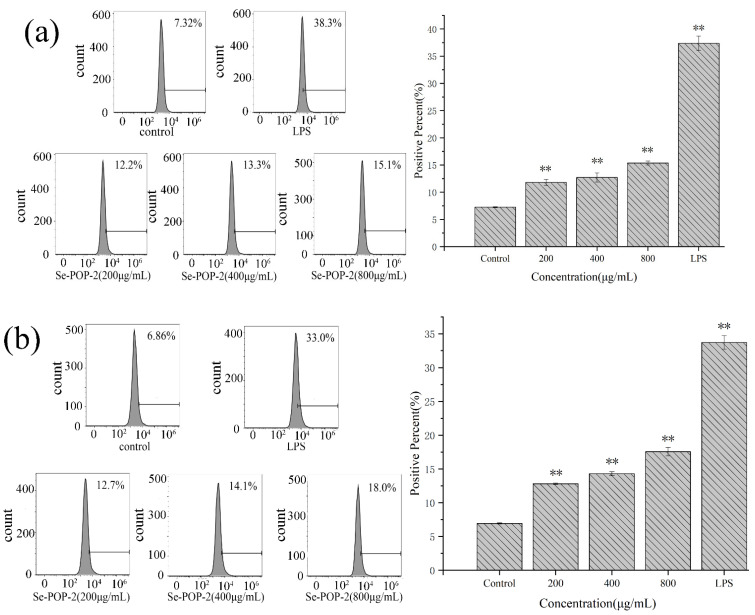
Effects of Se-POP-21 on the expression of CD80 (**a**) and CD86 (**b**) in RAW264.7 cells, as measured by flow cytometry. The data are expressed as mean ± SD; ** *p* < 0.01 compared with the control group.

After phagocytic absorption, macrophages express higher levels of costimulatory molecules, and play the role of antigen presentation, thus regulating the interaction between T lymphocytes and macrophages [38,39]. As shown in Figure 5, compared with the blank control group, the expression levels of CD80 and CD86 in RAW264.7 cells treated with Se-POP-21 (200, 400, 800 μg/mL) were significantly increased in a dose-dependent manner. Costimulatory signals play an essential role in T lymphocytes’ activation. Among them, CD80 and CD86 are two important molecules providing costimulatory signals. These are expressed on the surface of antigen-presenting cells, and bind to the ligand CD28 expressed on the surface of T lymphocytes to stimulate T lymphocytes’ activation and proliferation [40]. Se-POP-21 promotes the expression of CD08/CD86 in RAW264.7 cells better than *Pleurotus ferulae* polysaccharide [41], indicating that Se-POP-21 has apparent advantages in activating RAW264.7 cells. According to the report of Chen et al. [42], Se-POP-21 may regulate the expression of a variety of miRNAs, such as miR-155, which promotes macrophage activation by regulating NF-κB signaling to target different genes, thus activating costimulatory molecular signaling pathways. This may be one of the mechanisms by which Se-POP-21 enhances the body’s immune activity.

### 2.6. Effects on ROS Levels of RAW264.7 Cells

**Figure 6:**
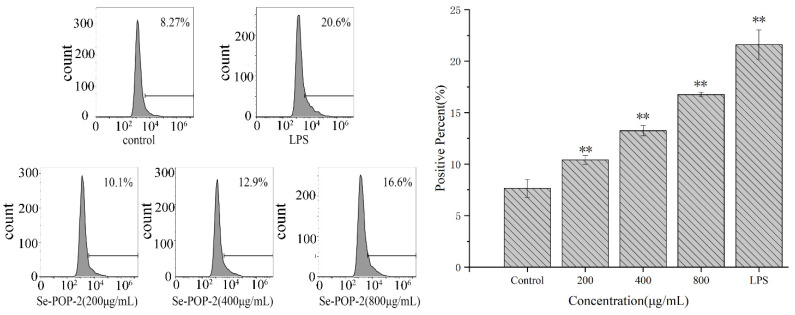
Effects of Se-POP-21 on the ROS levels of RAW264.7 cells, as measured by flow cytometry. The data are expressed as mean ± SD; ** *p* < 0.01 compared with the control group.

The DCFH-DA fluorescent probe was used to measure the levels of ROS in RAW264.7 cells. After entering the cells, the probe is hydrolyzed into non-fluorescent DCFH, and the ROS can oxidize DCFH into DCF with high fluorescence intensity. The fluorescence intensity is positively correlated with the intracellular ROS level [43]. Therefore, the fluorescence intensity is measured by flow cytometry to reflect the intracellular ROS level. As shown in Figure 6, after incubation with different concentrations of Se-POP-21 (200, 400, or 800 μg/mL), the intracellular ROS level gradually increased with the increase in Se-POP-21 concentration. Compared with the blank control group, the intracellular ROS levels of the Se-POP-21 groups increased significantly. Macrophages are the primary source of ROS, and the endogenous ROS can regulate major molecular signaling pathways, participate in the synthesis of inflammatory factors, and improve the phagocytosis ability of macrophages to kill pathogenic microorganisms [44].

### 2.7. Effect on NO Secretion of RAW264.7 Cells

**Figure 7:**
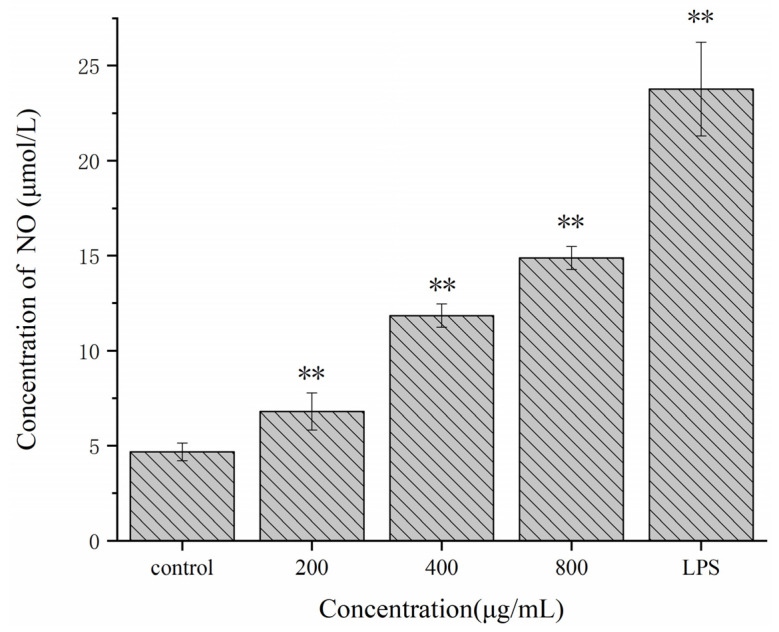
Effect of Se-POP-21 on the NO secretion of RAW264.7 cells. The data are expressed as mean ± SD; ** *p* < 0.01 compared with the control group.

The NO secreted by activated macrophages is considered to be an essential active mediator. It promotes tumor cell apoptosis and mediates biological reactions as a messenger molecule [45]. As shown in Figure 7, compared with the blank control group, Se-POP-21 significantly increased the NO secretion levels of RAW264.7 cells in a dose-dependent manner within the concentration range of 200–800 μg/mL. The promoting effect of Se-POP-21 on NO secretion from RAW264.7 cells was roughly the same as that of *Physalis alkekengi* polysaccharides [39], which may have been due to the similar monosaccharide composition of the two. However, the NO secretion level was still lower than that of the LPS-positive control group (the concentration of LPS is 1 μg/mL). This result suggests that Se-POP-21 may have the function of immune stimulation and inhibition of tumor cell proliferation.

### 2.8. Effects on the Secretion of TNF-α, IL-1β, and IL-6 by RAW264.7 Cells

**Figure 8:**
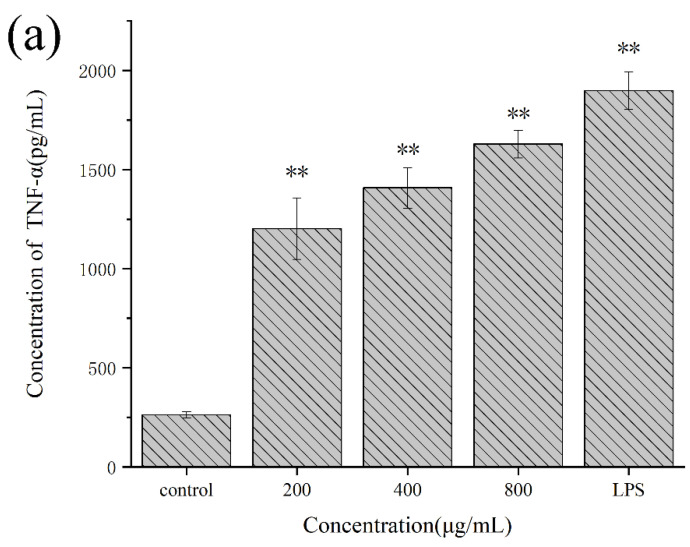

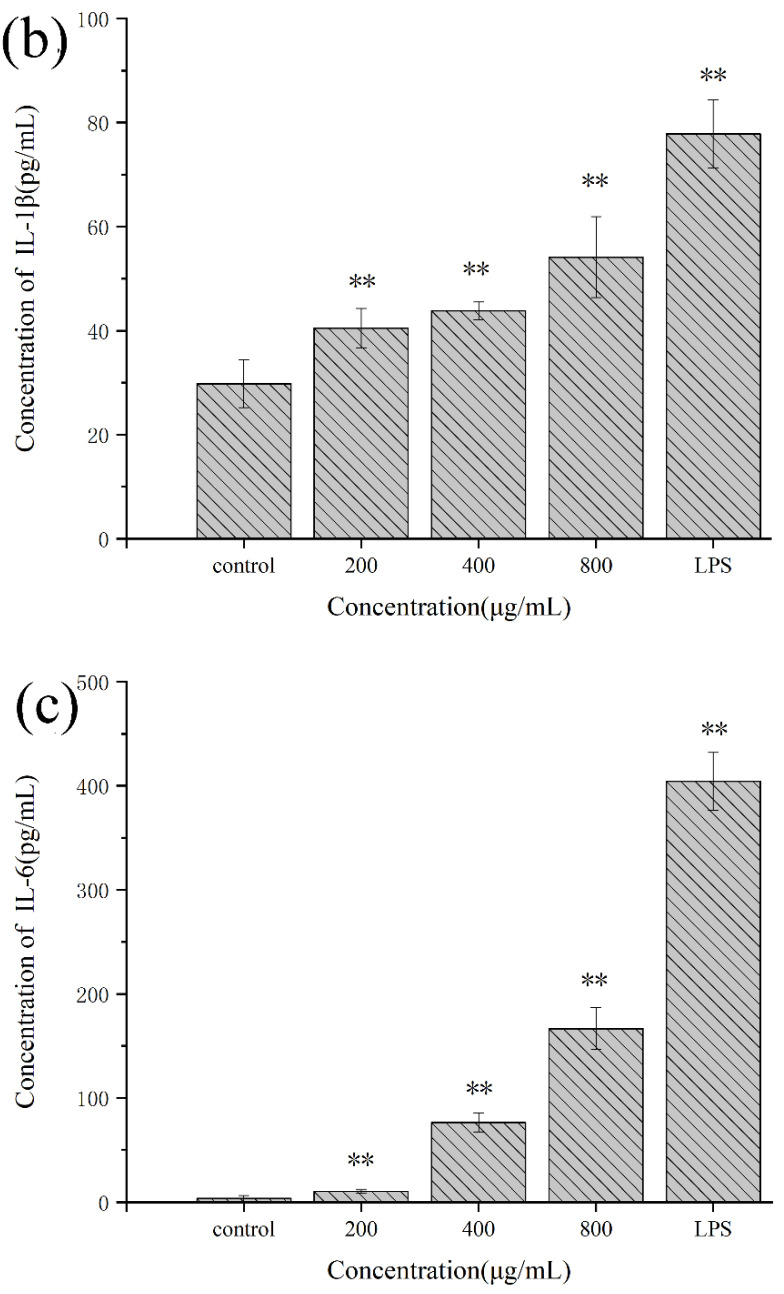
Effects of Se-POP-21 on the secretion of TNF-α (**a**), IL-1β (**b**), and IL-6 (**c**) by RAW264.7 cells. The data are expressed as mean ± SD; ** *p* < 0.01 compared with the control group.

As shown in Figure 8, the levels of the cytokines TNF-α, IL-1β, and IL-6 secreted by RAW264.7 cells treated with Se-POP-21 at various concentrations (200, 400, or 800 μg/mL) were significantly increased in a dose-dependent manner. Cytokines produced by activated immune cells can promote the proliferation and differentiation of target cells, induce receptor expression, and play an essential role in regulating the body’s immune response [46]. A variety of fungal polysaccharides have been confirmed to induce the secretion of cytokines [47]. TNF-α is a cytokine secreted mainly by activated macrophages and lymphocytes, and can initiate immune regulation, improve the activity of immune cells, and directly kill tumor cells [48]. IL-1β is produced by activated macrophages, and stimulates the proliferation of T and B lymphocytes [49]. IL-6 is a pleiotropic cytokine that can promote the growth and differentiation of bone-marrow-derived cells and participate in the body’s immune defense [50]. These results indicate that Se-POP-21 may enhance the body’s immune activity by promoting the secretion of TNF-α, IL-1β, and IL-6 by RAW264.7 cells. At the same concentrations, the promoting effect of Se-POP-21 on the cytokines’ secretion was significantly better than that of *Sinonovacula constricta* polysaccharides. However, the impact of Se-POP-21 on the phagocytosis activity was not as good as that of *Sinonovacula constricta* polysaccharides [51], indicating that different polysaccharides have different advantages. 

### 2.9. Regulation of NF-κB Protein Expression in RAW264.7 Cells

**Figure 9:**
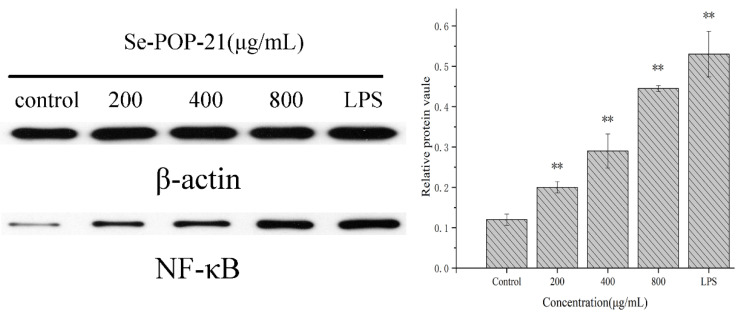
Effect of Se-POP-21 on the NF-κB protein expression in RAW264.7 cells. The data are expressed as mean ± SD; ** *p* < 0.01 compared with the control group.

NF-κB is a vital transcription factor widely present in the immune process. Functional NF-κB binding sites exist in promoters and enhancers of many genes. It regulates many transcription genes in immune and inflammatory responses [52]. For example, Zha et al. [53] found that *Laminaria japonica* polysaccharide can initiate the NF-κB signaling pathway and regulate the expression of TNF-α and IL-6 mRNA, thereby promoting the expression of corresponding cytokines. In addition, Sun et al. [54] found that NF-κB activation is associated with polysaccharides from the roots of *Actinidia eriantha*. As shown in Figure 9 (original images for blots are shown in the Supplementary Materials), as the concentration of Se-POP-21 increases, the expression level of NF-κB in RAW264.7 cells gradually increases. In summary, this is a possible mechanism of Se-POP-21’s immunomodulatory activity: A large amount of free mannose at the end of the Se-POP-21 branched chain binds to the mannose receptor—an important pattern recognition receptor on macrophages—activating a specific signal transduction pathway, stimulating the expression of NF-κB proteins, promoting the secretion of cytokines and, thus, playing a role in immunomodulatory activity [55].

## 3. Materials and Methods

### 3.1. Materials

Se-POP-21 was extracted and purified from selenium-enriched *Pleurotus ostreatus* using the method outlined in our previous report [22]. The murine monocyte macrophage RAW264.7 was provided by Hunan Agricultural University (Changsha, China). The Cell Counting Kit-8 was purchased from New Cell & Molecular Biotech Co., Ltd. (Suzhou, China). Neutral red dye solution and ELISA kits were purchased from Sangon Biotech (Shanghai) Co., Ltd. (Shanghai, China). Nitric oxide detection kits, lipopolysaccharide (LPS), and reactive oxygen species assay kits were purchased from Beyotime Biotechnology Co., Ltd. (Shanghai, China). CD80 and CD86 antibodies (FITC) were purchased from Sino Biological Inc. (Beijing, China). All other reagents used in the study were analytically pure.

### 3.2. Structure Analysis of Se-POP-21

#### 3.2.1. Analysis of Methylation

Methylation analysis is one of the most classical and essential methods for the structural analysis of polysaccharides, and is mainly used to analyze and determine the connection mode of glycosides [24]. All of the free hydroxyl groups in the polysaccharide were methylated and then hydrolyzed to obtain partially methylated monosaccharides. The monosaccharides were reduced and acetylated to produce partially methylated alditol acetates (PMAAs). Finally, the type and quantity of the partially methylated derivatives were determined by GC–MS, and the connection of monosaccharide residues could be inferred. Methylation analysis drew on the method reported by Carbonero et al. [56]. Briefly, dried Se-POP-21 (10 mg, about 2.5 × 10^−3^ mmol) and NaOH (40 mg, 1 mmol) were dissolved in 3 mL of anhydrous DMSO with ultrasound, and then 2.5 mL of CH_3_I was added for methylation reaction for 2 h. The reaction was terminated by adding 2 mL of ultrapure water and extracted by CHCl_3_, and the organic phase was washed with water 4 times. Then, the methylated polysaccharide was evaporated to dryness and cleaned with methyl alcohol 3 times. The product was treated with 3 mL of 2 M TFA (trifluoroacetic acid), hydrolyzed for 90 min at 110 °C, evaporated to dryness, and washed twice with methyl alcohol. Next, 6 mL of double-distilled water and 90 mg of NaBH_4_ (2.38 mmol) were added to react for 8 h, and the pH value was adjusted to 7 by adding glacial acetic acid. The reduction product was evaporated to dryness and dried at 100 °C. Then, 3 mL of acetic anhydride was added for acetylation at 100 °C for 1 h. After that, the acetylated product was washed with methylbenzene 5 times, dissolved in 3 mL of CH_2_Cl_2_, and dried with anhydrous sodium sulfate. Finally, the prepared sample was analyzed using a GCMS-QP 2010 Plus spectrometer (the column type was RXI-5 SIL MS, 30 × 0.25 × 0.25) (Shimadzu, Japan).

#### 3.2.2. Analysis of NMR

A total of 50 mg of Se-POP-21 was dissolved in 500 μL of D_2_O and lyophilized. The above steps were repeated twice to replace hydrogen with deuterium [54]. ^1^H NMR, ^13^C NMR, COSY, NOESY, HSQC, and HMBC spectra of Se-POP-21 were recorded at 25 °C using a Bruker 400 MHz NMR spectrometer (Rheinstetten, Germany).

### 3.3. Immunomodulatory Activities of Se-POP-21

#### 3.3.1. RAW264.7 Cells’ Viability

The RAW264.7 cells were inoculated onto 96-well plates at a density of 1 × 10^5^ cells/mL (100 μL of cell solution per well), and placed in an incubator (37 °C, 5% CO_2_) for 24 h. Then, the cells were cultured with 100 μL of different concentrations of Se-POP-21 solution (0, 50, 100, 200, 400, or 800 μg/mL) and incubated for 24 h. After incubation, the original medium was discarded, and 100 μL of 10% CCK-8 solution was added to each well. After incubating for 1 h, the absorbance of the supernatant of each well was measured at 450 nm using a microplate reader (Thermo, Waltham, MA, USA).

#### 3.3.2. Phagocytosis Capacity of RAW264.7 Cells

The phagocytic ability of RAW264.7 cells was measured by the neutral red method [55]. The RAW264.7 cells were inoculated into 96-well plates with 5 × 10^5^ cells/mL and incubated for 24 h. Then, the cells were stimulated with 100 μL of various concentrations of Se-POP-21 solution (0, 200, 400, or 800 μg/mL) or LPS (a potent immune-cell activator) solution (1 μg/mL), and continued to be incubated for 24 h. After that, 1% neutral red PBS solution was added to each well (100 μL per well) and set for 2 h. Next, the neutral red solution was removed, and each well was washed 3 times with PBS to eliminate the effects of free neutral red. Then, the cells in each well were incubated with 100 μL of cell lysis buffer (absolute ethanol: acetic acid = 1:1) for 5 h at 4 °C to destroy the cells, and the absorbance was measured at 540 nm. 

#### 3.3.3. Analysis of Phenotypic Characterization

The RAW264.7 cells were seeded in 12-well plates at a density of 2 × 10^5^ cells/mL and incubated for 24 h. The original medium was removed, and various concentrations of Se-POP-21 solution (0, 200, 400, or 800 μg/mL) or LPS solution (1 μg/mL) were added to each well. After incubation for 24 h, the cells were collected and incubated with murine CD80/CD86 antibody (FITC) and analyzed by flow cytometry (Beckman Coulter, Brea, CA, USA) [37]. 

#### 3.3.4. Measurement of ROS Production by RAW264.7 Cells

The content of reactive oxygen species (ROS) in RAW264.7 cells was determined by the DCFH-DA fluorescence probe method [38]. The methods of RAW264.7 cells’ inoculation and treatment were the same as described in3.3.3. After incubation for 24 h, the supernatant was removed, and 100 μL of DCFH-DA solution was added to each well. The supernatant was discarded after incubating for 20 min. Then, the cells were washed with PBS 3 times to remove free DCFH-DA, suspended in 500 μL of PBS, and analyzed by flow cytometry.

#### 3.3.5. Measurement of NO Secretion by RAW264.7 Cells 

The RAW264.7 cells were inoculated into 96-well plates at a density of 5 × 10^5^ cells/mL and incubated for 24 h. Then, the cells were cultured with 100 μL of different concentrations of Se-POP-21 solution (0, 200, 400, or 800 μg/mL) or LPS solution (1 μg/mL), and then incubated for 24 h. The NO level in the supernatant was detected using a nitric oxide assay kit containing Griess reagent [56].

#### 3.3.6. Enzyme-Linked Immunosorbent Assay

The RAW264.7 cells were seeded in 96-well plates (1 × 10^5^ cells/well), incubated for 24 h, and stimulated with 100 μL of different concentrations of Se-POP-21 solution (0, 200, 400, or 800 μg/mL) or LPS solution (1 μg/mL), and then continued to be incubated for 24 h. The supernatant of the cells was collected, and the contents of TNF-α, IL-1β, and IL-6 were measured using their corresponding ELISA kits.

#### 3.3.7. Western Blotting Assay

The RAW264.7 cells were loaded into 6-well plates (2 × 10^5^ cells/well) and incubated for 24 h. After that, the original media were changed to new media with different concentrations of Se-POP-21 solution (0, 200, 400, or 800 μg/mL) or LPS solution (1 μg/mL) and incubated for 24 h continuously. Then, the supernatant was removed, and the cells were washed with iced PBS once. After that, 200 μL of RIPA lysate was added to each well, and the cell suspension was collected. After lysis on ice for 10 min, the cells were centrifuged at 4 °C at 12,000 RPM for 15 min, and the supernatant was collected. The protein concentration was determined using the BCA protein quantification kit. The proteins were separated by polyacrylamide gel electrophoresis (SDS–PAGE), transferred to a nitrocellulose (NC) filter membrane, and sealed with 5% skimmed milk powder prepared with PBST for 90 min. The primary antibody was diluted proportionally (anti-NF-κB 1:1000; anti-β-actin 1:5000) and incubated with the membrane at room temperature for 90 min. The HRP-conjugated secondary antibody was diluted (1:5000) and incubated with the membrane at room temperature for 90 min. The target proteins were detected using ECL Western blot detection reagents (Cwbiotech, Beijing, China).

### 3.4. Statistical Analysis

All experiments were repeated three times, and the experimental results were expressed as the mean ± standard deviation (SD). The data between different groups were represented by Student’s *t*-test. A significance level of *p* < 0.05 represents a significant difference.

## 4. Conclusions

In this study, the primary structural unit formula of Se-POP-21 was successfully resolved by methylation analysis and NMR experiments. The backbone of Se-POP-21 is →[2,6)-α-D-Galp-(1→6)-α-D-Galp-(1]_4_→2,4)-β-L-Arap-(1→[2,6)-α-D-Galp-(1→6)-α-D-Galp-(1]_4_→, composed of a large amount of galactose. Therefore, Se-POP-21 can be approximated as a novel galactose glycan. The previous study showed that the selenium content of Se-POP-21 is 5.31 μg/g [22]. Due to the low selenium content, this study could not accurately describe the position of selenium in the structure of Se-POP-21. However, according to the FTIR spectrum obtained from previous studies, it can be inferred that selenium may be linked to Se-POP-21 in the form of selenious acid through the Se-O-C bond. Biological experiments show that Se-POP-21 could promote the proliferation and phagocytosis of RAW264.7 cells, activate the expression of the NF-κB protein, and promote the secretion of NO, ROS, TNF-α, IL-1β, and IL-6 by RAW264.7 cells. The above results indicate that Se-POP-21 can effectively activate RAW264.7 cells. However, the mechanism of Se-POP-21’s immunomodulatory activity and the relationship between structure and biological activity need further study.

## Figures and Tables

**Table 1:** GC–MS data of methylation analysis of Se-POP-21.

Methylated Sugar	Molar Ratio	Mass Fragments (m/z)	Retention Time	Type of Linkage
2,3,4,6-Me_4_-Manp	0.325	43, 71, 87, 101, 117, 129, 145, 161, 205	15.35	Manp-(1→
2,3,4,6-Me_4_-Galp	0.012	43, 71, 87, 101, 117, 129, 145, 161, 205	16.03	Galp-(1→
2,4-Me_2_-Arap	0.016	43, 59, 101, 117, 129, 174, 201, 233	17.99	→3)-Arap-(1→
2,3-Me_2_-Arap	0.041	43, 87, 101, 117, 129, 161, 189, 233	19.02	→4)-Arap-(1→
2,3,4,-Me_3_-Galp	0.280	43, 87, 99, 101, 117, 129, 161, 189, 233	20.17	→6)-Galp-(1→
3-Me-Arap	0.039	43, 71, 87, 99, 129, 159, 189,233	20.87	→2,4)-Arap-(1→
3,4,-Me_2_-Galp	0.287	43, 71, 87, 99, 129, 173, 189, 233	22.90	→2,6)-Galp-(1→

**Table 2:** The chemical shifts and analysis results of ^13^C and ^1^H of the residues.

Residues	H1/C1	H2/C2	H3/C3	H4/C4	H5a/C5	H5b/C5	H6a/C6	H6b/C6
A	4.74	4.03	3.56	3.33	3.54		3.70	3.87
β-D-Manp-(1→	101.62	70.32	72.55	66.78	76.21		61.07	61.07
B	4.89	3.77	3.54	3.33	3.87	3.71		
→4)-β-L-Arap-(1→	98.15	66.89	66.51	76.26	61.04	61.04		
C	4.94	3.79	3.89	4.05	3.58		3.86	3.65
→6)- α-D-Galp-(1→	97.66	68.25	68.52	68.95	70.83		66.66	66.66
D	4.86	3.64	4.24	3.49	3.88	3.71		
→2,4)- β-L-Arap-(1→	98.68	78.11	65.15	78.78	61.04	61.04		
E	5.06	3.90	4.14	3.96	3.84		3.64	3.86
→2,6)- α-D-Galp-(1→	98.34	76.97	68.96	69.35	69.53		66.65	66.65

## Data Availability

Not applicable.

## Supplementary Materials

The following supporting information can be downloaded at: https://www.mdpi.com/article/10.3390/molecules27082591/s1. The Supplementary Materials contain the mass spectrograms of PMAA and the original images for blots. Figure S1: The EI–MS fragmentation spectrum of Manp-(1→; Figure S2: The EI–MS fragmentation spectrum of Galp-(1→; Figure S3: The EI–MS fragmentation spectrum of →3)-Arap-(1→; Figure S4: The EI–MS fragmentation spectrum of →4)-Arap-(1→; Figure S5: The EI–MS fragmentation spectrum of →6)-Galp-(1→; Figure S6: The EI–MS fragmentation spectrum of →2,4)-Arap-(1→; Figure S7: The EI–MS fragmentation spectrum of →2,6)-Galp-(1→; Figure S8: The original image for the Western blot of β-actin; Figure S9: The original image for the Western blot of NF-κB.
