# Supplementary material for: The Main Structural Unit Elucidation and Immunomodulatory Activity In Vitro of a Selenium-Enriched Polysaccharide Produced by Pleurotus ostreatus
Source: Molecules. 2022 Apr 18;27(8):2591. doi: 10.3390/molecules27082591 (PMC9027278; doi:10.3390/molecules27082591)
Supplement: Supplementary file 1 [file molecules-27-02591-s001.zip › molecules-1659390-supplementary.pdf]

Supplementary Materials

The main structural unit elucidation and immunomodulatory activity *in vitro* of a selenium-enriched polysaccharide produced by *Pleurotus ostreatus*

De Wang, Jiahui Wang, Hui Liu, Meng Liu, Yanjing Yang[*], Shian Zhong[*]

College of Chemistry and Chemical Engineering, Central South University, Changsha 410083, China

*Corresponding author:

College of Chemistry and Chemical Engineering, Central South University, Changsha, Hunan, 410083, China

Tel: +86 0731 88879616; Fax: +86 0731 88879616;

E-mail: yangyanjing@csu.edu.cn (Yanjing Yang)

E-mail: zhongshian@aliyun.com (Shian Zhong)

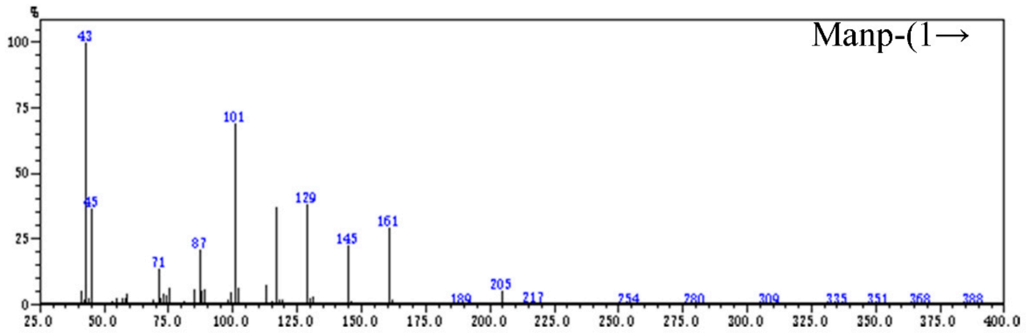

Figure S1. The EI-MS fragmentation spectrum of the Manp-(1→

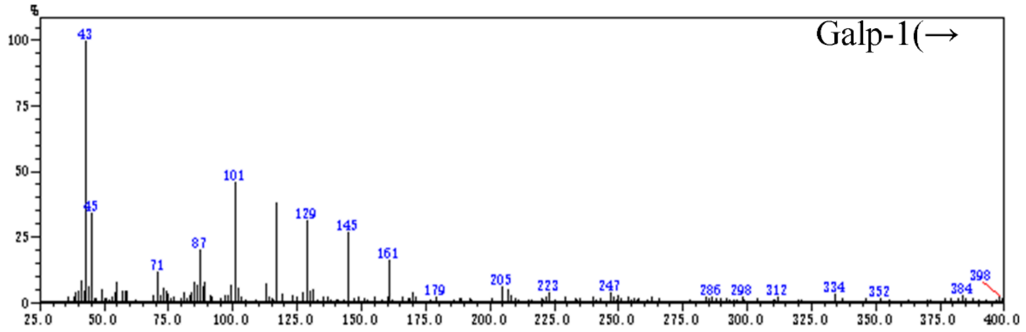

Figure S2. The EI-MS fragmentation spectrum of the Galp-(1→

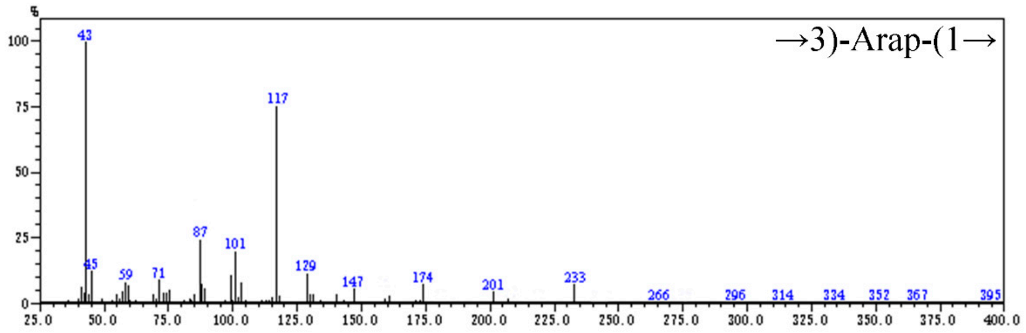

Figure S3. The EI-MS fragmentation spectrum of the →3)-Arap-(1→

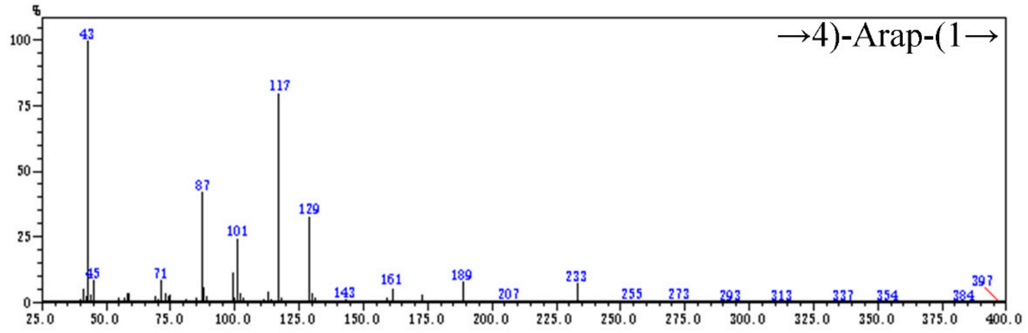

Figure S4. The EI-MS fragmentation spectrum of the →4)-Arap-(1→

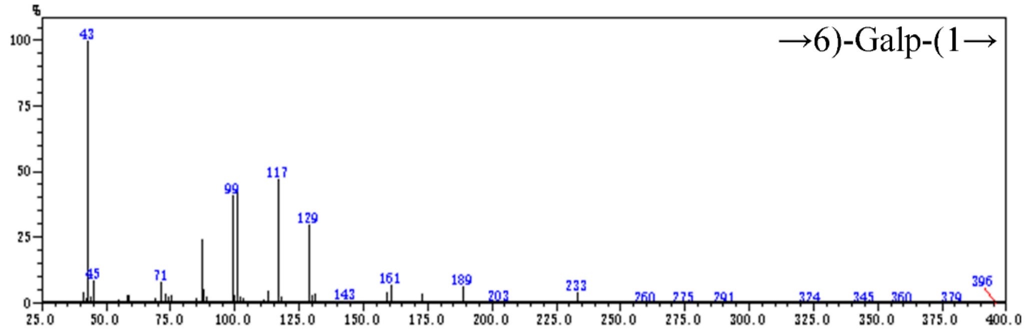

Figure S5. The EI-MS fragmentation spectrum of the →6)-Galp-(1→

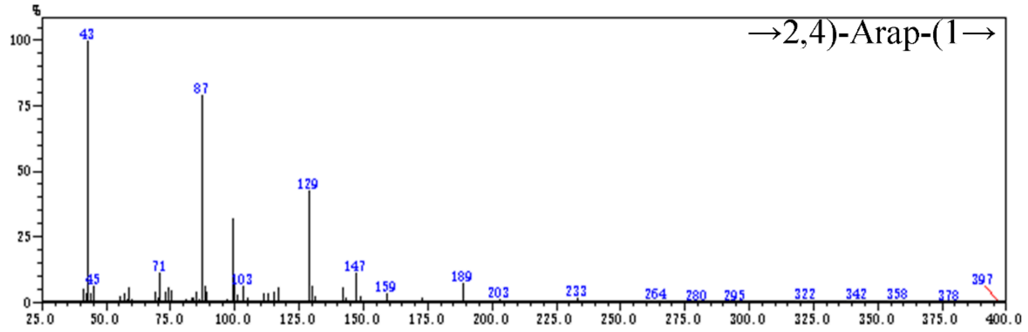

Figure S6. The EI-MS fragmentation spectrum of the →2,4)-Arap-(1→

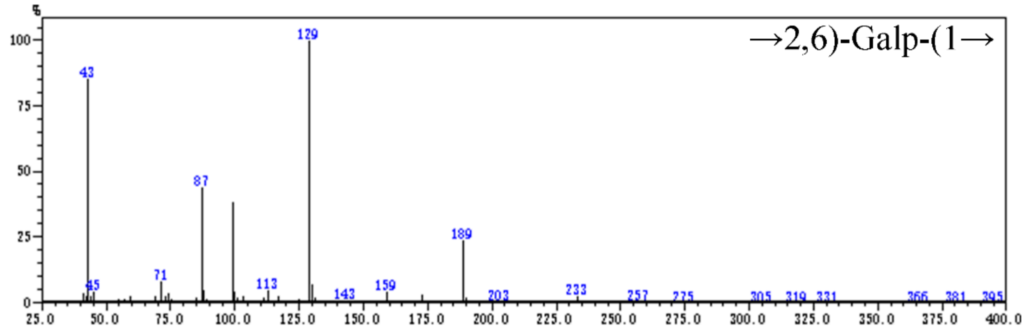

Figure S7. The EI-MS fragmentation spectrum of the →2,6)-Galp-(1→

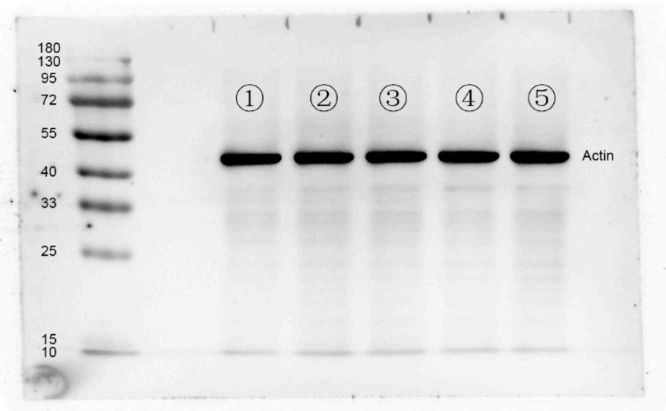

Figure S8. The original image for western blot of β-actin

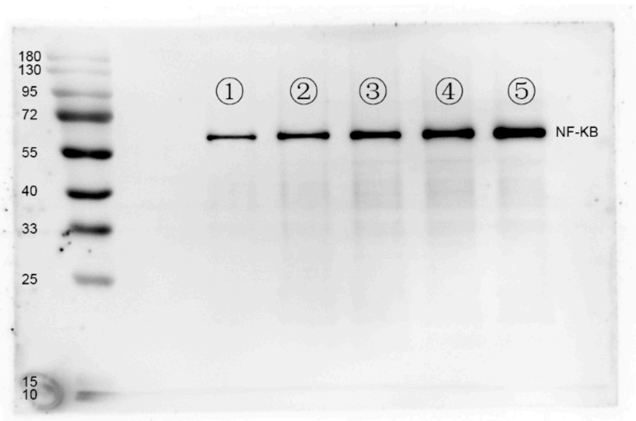

Figure S9. The original image for western blot of NF-κB
